# Supplementary material for: Transcription factor IRF-5 regulates lipid metabolism and mitochondrial function in murine CD8+ T-cells during viral infection
Source: EMBO J. 2025 Jun 10;44(15):4280–300. doi: 10.1038/s44318-025-00485-2 (PMC12316916; doi:10.1038/s44318-025-00485-2)
Supplement: Supplementary file 1 — Appendix [file 44318_2025_485_MOESM1_ESM.pdf]

Appendix for **“Transcription factor IRF-5 regulates lipid metabolism and mitochondrial function in murine CD8+ T-cells during viral infection”**

Linh Thuy Mai[1], Sharada Swaminathan[1], Trieu Hai Nguyen[1], Etienne Collette[2], Tania Charpentier[1], Liseth Carmona-Pérez[1], Hamza Loucif[3], Alain Lamarre[1], Krista M. Heinonen[1], David Langlais[2], Jörg H. Fritz[2], and Simona Stäger[1]

[1] Centre Armand-Frappier Santé Biotechnologie, Institut National de la Recherche Scientifique, Laval (QC), Canada

[2] Dahdaleh Institute of Genomic Medicine, Department of Microbiology and Immunology, Department of Human Genetics, McGill University, Montreal, QC H3A 0G1, Canada.

[3] Department of Microbiology and Immunology, McGill University, Montréal, Canada; FOCiS Centre of Excellence in Translational Immunology (CETI), Montréal, Canada; McGill University Research Centre on Complex Traits (MRCCT), Montréal, Canada. Department of Physiology, McGill University, Montreal, QC H3A 0G1, Canada.

Table of Contents

Table of Contents	Page
Appendix Figure S1	1
Appendix Figure S2	2
Appendix Figure S3	3
Appendix Figure S4	4
Appendix Figure S5	5
Appendix Figure S6	6
Appendix Figure S7	7
Appendix Figure S8	8
Appendix Table S1	9

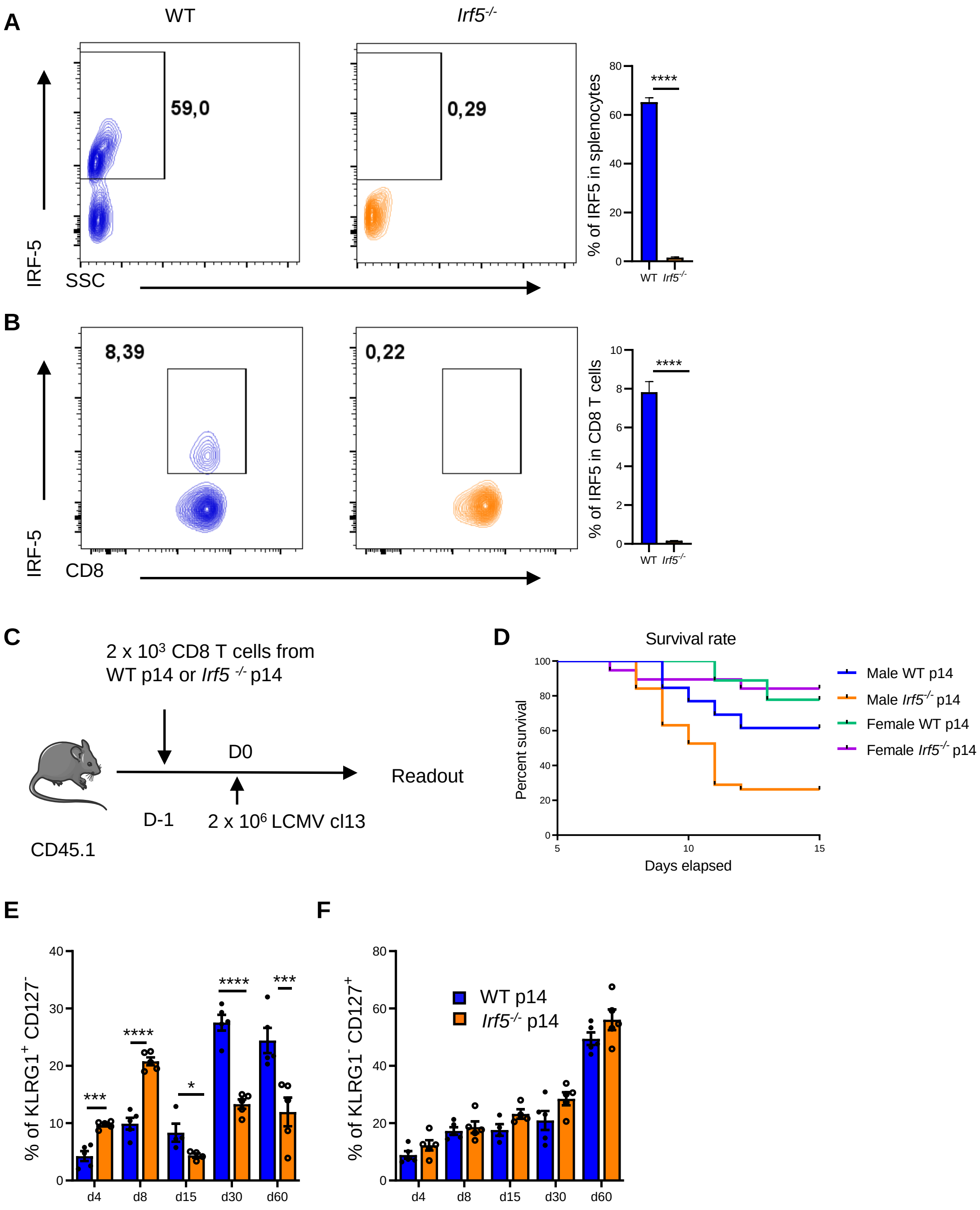

Appendix Figure S1. (A-B) Representative FACS plots and graphs representing IRF-5 expression by total splenocyte (A) and CD8 T cells (B) from WT (*Irf5*$^{f/f}$ *x CMV- cre*$^{-}$) and *Irf5*$^{-/-}$ (*Irf5*$^{f/f}$ *x CMV- cre*$^{+}$) p14 transgenic mice. (C) Experimental set-up for adoptive transfer experiments described throughout the manuscript. (D) Graph illustrates the survival rate of male or female CD45.1 recipient mice adoptively transferred ~~or not~~ with WT and *Irf5*$^{-/-}$ p14 CD8 T cells over the course of LCMV Cl13 infection. Graphs show (E) the percentage of KLRG1^{+} CD127^{-} and (F) the percentage of CD44^{+}CD127^{+} WT and *Irf5*$^{-/-}$ p14 CD8 T cells found in the spleen of recipient female mice over the course of infection.

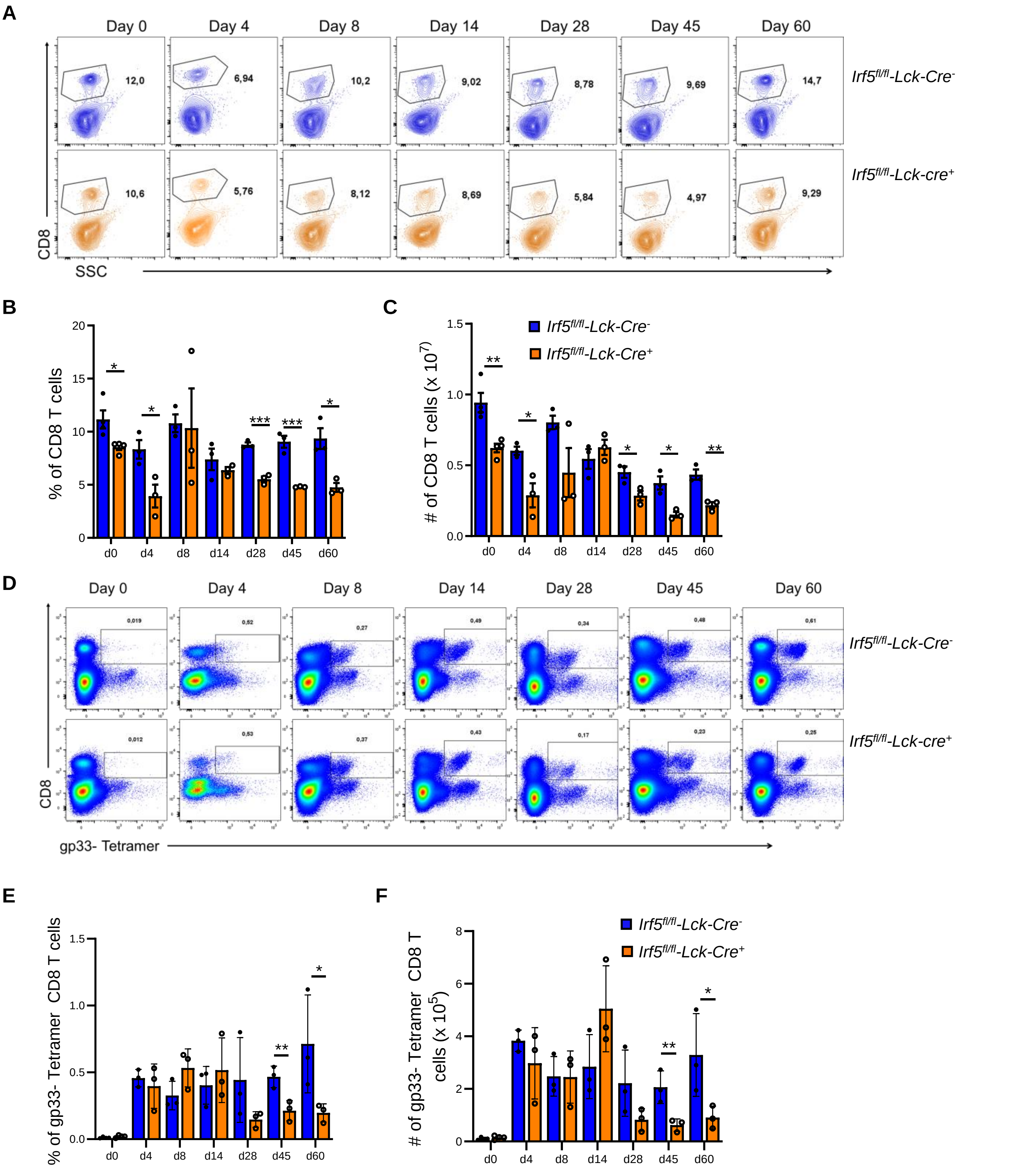

Appendix Figure S2. *Irf5f/fx Lck- Cre-* (WT) and *Irf5f/fx Lck- Cre+* (Lck-cre+) mice were infected intravenously with 2 × 10^6 PFU LCMV Cl13 and euthanized at various time points p.i.. Graphs show (A) representative FACS plots for CD8 T cell staining, and (B) the percentages and (C) absolute numbers of CD8 T cells found in the spleen over the course of infection. Graphs illustrate (D) representative flow cytometry plots for gp33-tetramer and CD8 staining, and (E) the percentages and (F) the absolute numbers of gp33-tetramer+ CD8 T cells found in the spleen throughout infection.

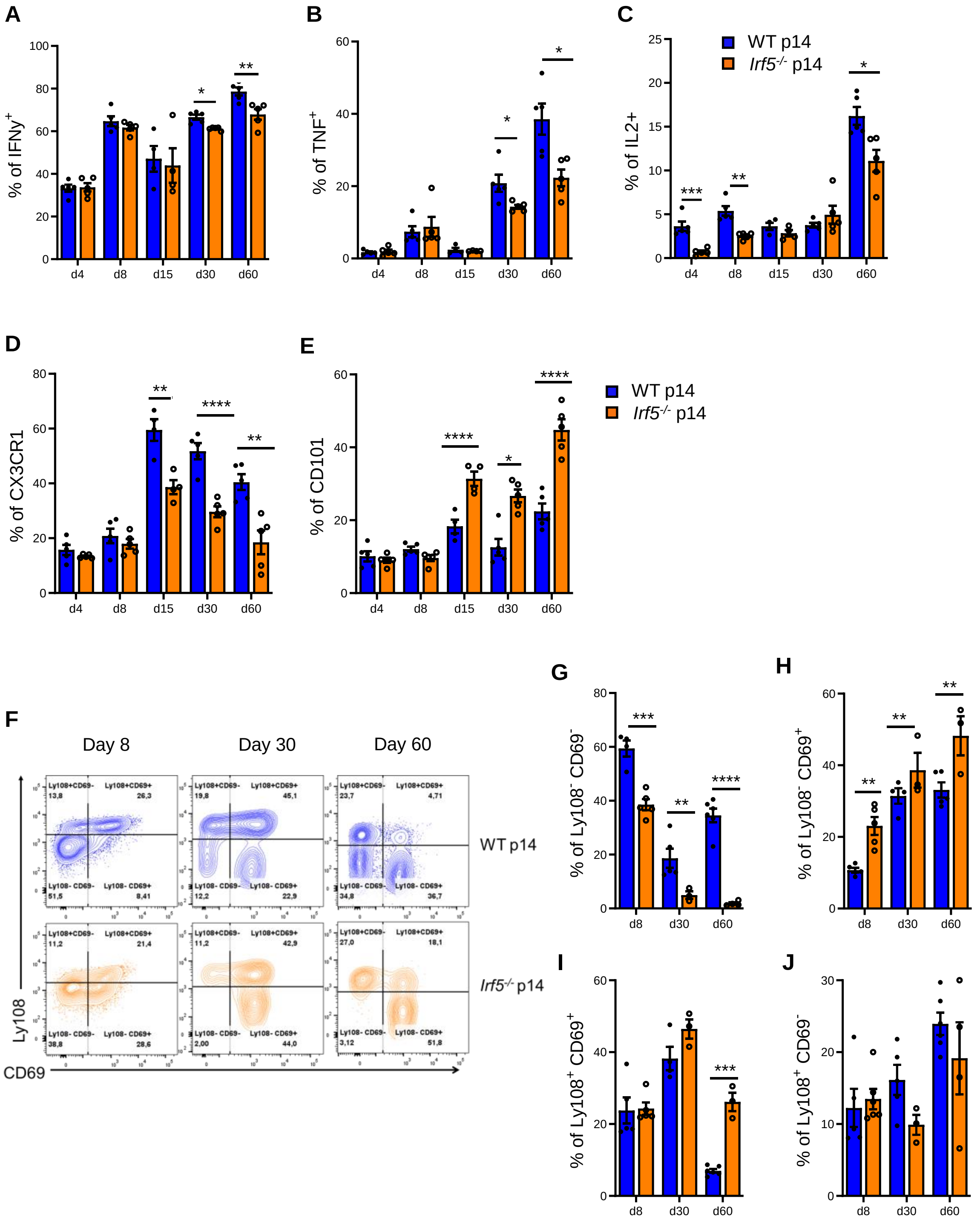

Appendix Figure S3. CD8 T cells from female WT or *Irf5*$^{-/-}$ p14 mice were adoptively transferred into CD45.1 recipient mice one day prior to intravenous infection with 2 × 10^6 PFU LCMV Cl13. Mice were euthanized at various time points after infection. Graphs show the frequency of (A) IFNγ$^+$, (B) TNF$^+$, and (C) IL-2$^+$ CD8 T cells upon *in vitro* restimulation with the gp33 peptide; and the percentage of (D) CX3CR1$^+$ and (E) CD101$^+$ splenic CD8 T cells. (F) Representative FACS plots for the expression of Ly108 and CD69 over the course of LCMV Cl13 infection in adoptively transferred WT and *Irf5*$^{-/-}$ p14 CD8 T cells. Frequencies of (G) Tex^{int}, (H) Tex^{term} (I) Tex^{Prog1}, and (J) Tex^{Prog2} WT and *Irf5*$^{-/-}$ p14 CD8 T cells.

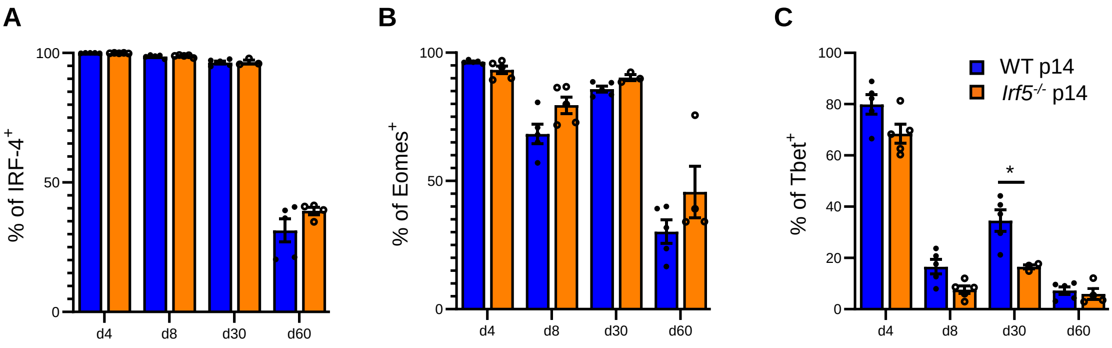

Appendix Figure S4. Graphs illustrate the frequency of (A) IRF-4⁺, (B) Eomes⁺, and (C) T-bet⁺ WT and *Irf5*$^{-/-}$ p14 CD8 T cells found in the spleen of recipient mice at various time points after LCMV Cl 13 infection.

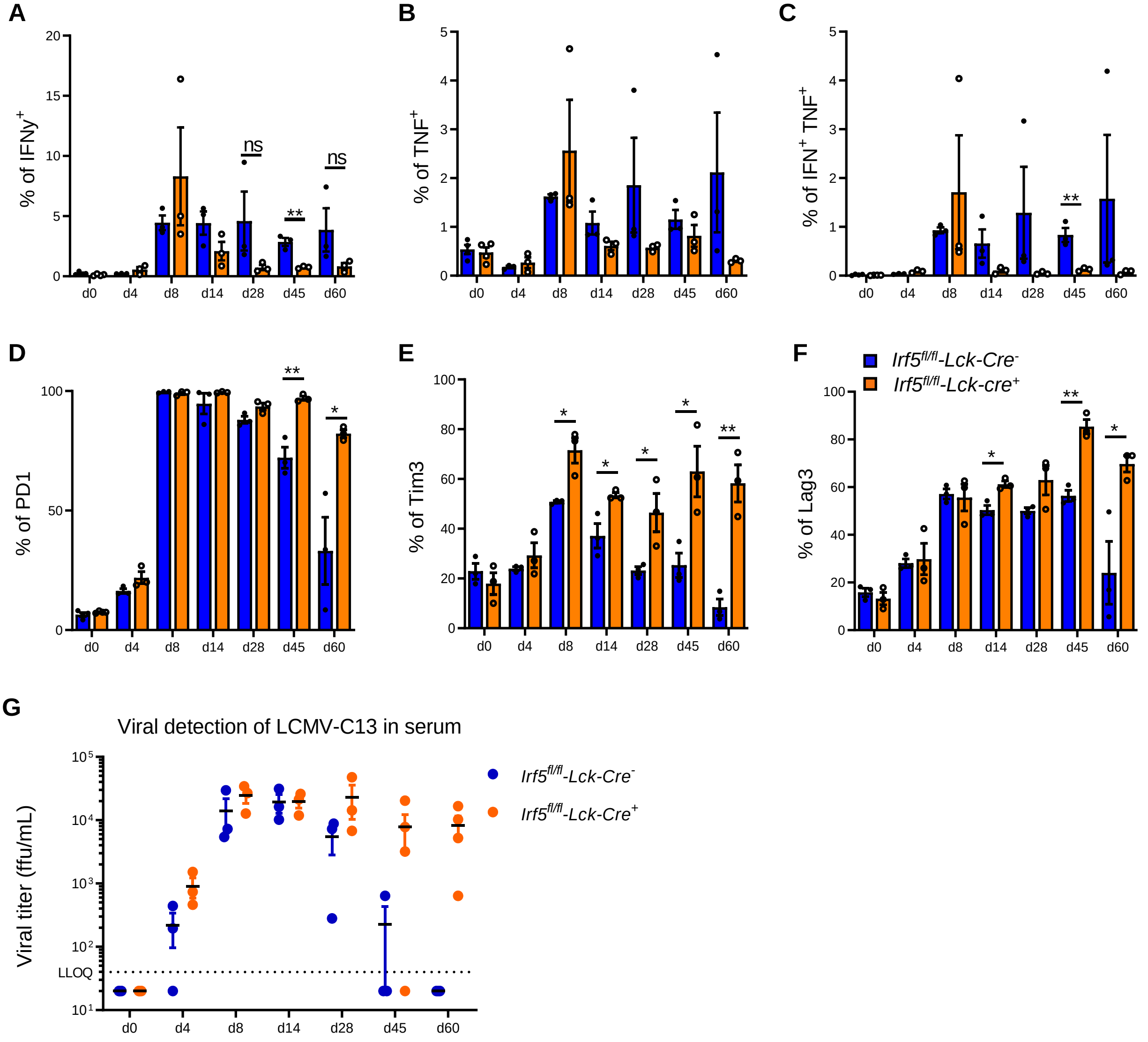

Appendix Figure S5. *Irf5*$^{f/f}$*x Lck- Cre*$^{-}$ (WT) and *Irf5*$^{f/f}$*x Lck- Cre*$^{+}$ (Lck-cre^{+}) mice were infected intravenously with 2 × 10^6 PFU LCMV Cl13 and euthanized at various time points p.i.. Graphs show the frequency of (A) IFNγ^{+}, (B) TNF^{+}, and (C) IFNγ^{+} TNF^{+} CD8 T cells upon *ex vivo* restimulation with the gp33 peptide; the percentage of gp33-tetramer^{+} splenic CD8 T cells expressing (D) PD-1, (E) TIM-3, and (F) LAG-3 over the course of infection. (G) Viral titers were assessed in the serum of infected mice until d60 p.i.

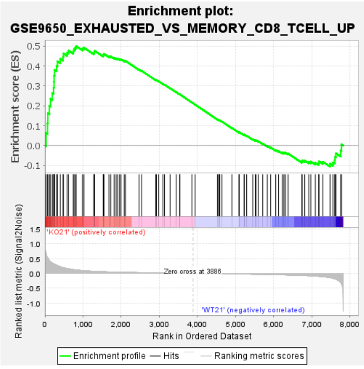

Appendix Figure S6. Enrichment score for exhaustion signature genes in WT and *Irf5*$^{-/-}$ p14 CD8 T cells isolated from female and male recipient mice at d21 p.i..

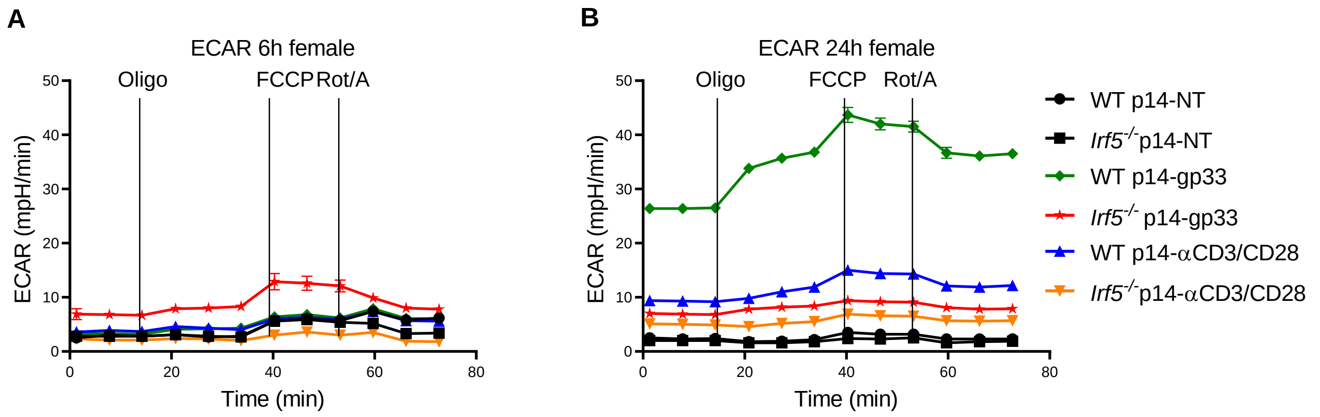

Appendix Figure S7. WT and *Irf5*$^{-/-}$ p14 CD8 T cells purified from female mice were cultured *in vitro* in the presence or absence of the gp33 peptide or anti-CD3/CD28, and the extracellular acidification rates over time were measured using the Seahorse XFe-96 analyzer at (A) 6h and (B) 24h after stimulation.

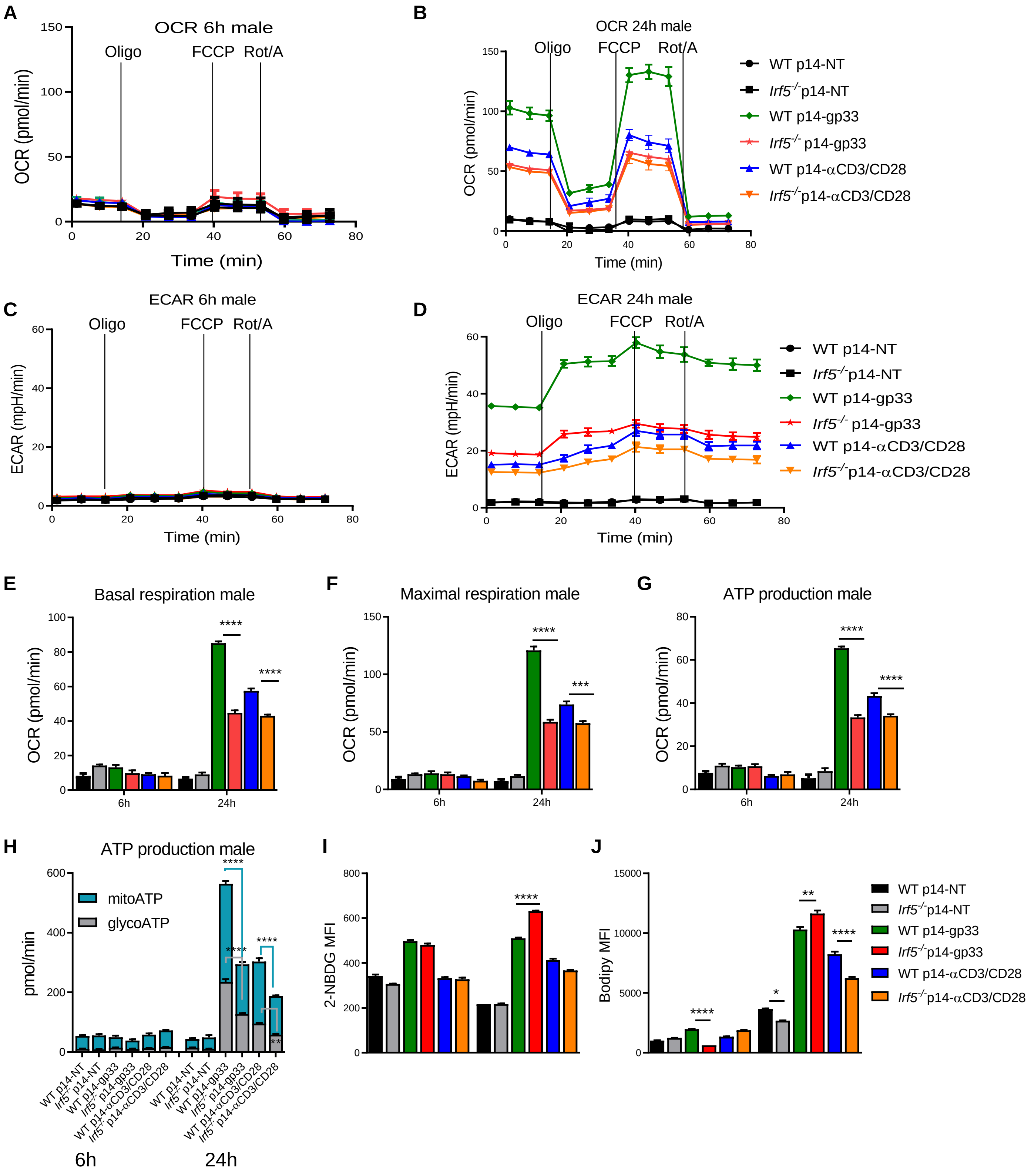

Appendix Figure S8. WT and *Irf5*$^{-/-}$ p14 CD8 T cells purified from male mice were cultured *in vitro* in the presence or absence of the gp33 peptide or anti-CD3/CD28, and the mitochondrial respiratory capacity was measured using the Seahorse XFe-96 analyzer. Graphs illustrate (A, B) the representative oxygen consumption rates (OCR) over time at (A) 6h and (B) 24h after stimulation; (C,D) the extracellular acidification rates (ECAR) over time at (C) 6h and (D) 24h after stimulation; (E) the basal and (F) maximal respiration, and (G) the ATP production at 6h and 24h after stimulation with the gp33 peptide or with CD3/CD28; (H) the ATP production rate; and (I) the glucose (measured using the fluorescent glucose analog 2-NBDG) and (J) the fatty acid uptake capacity (quantified using bodipy FLC_{16} fluorescence intensity).

Appendix Table S1: Top 45 DEG genes

EnsemblID	Gene	log2Fold Change	pvalue	padj	Day_21_male _WT1	Day_21_male _WT4	Day_21_ female_ WT2	Day_21_ female_ WT3	Day_21_male _KO1	Day_21_m ale_KO4	Day_21_female _KO2	Day_21_female _KO3
ENSMUSG00000056999.15	Ide	-1.04486	1.43E-19	2.39E-15	6811.519	6758.109	5842.436	5333.552	3636.681	2893.239	2643.116	2836.387
ENSMUSG00000040565.8	Btaf1	-1.00293	1.16E-16	9.64E-13	4063.14	3717.092	3366.403	2764.195	1911.418	1570.874	1628.447	1836.976
ENSMUSG00000005262.13	Ufd1	0.666573	2.82E-12	1.57E-08	1097.398	1078.815	938.7247	998.2274	1582.322	1508.37	1635.359	1807.889
ENSMUSG00000019982.15	Myb	1.347529	6.31E-10	2.63E-06	271.0931	232.4008	228.4286	192.3495	732.9874	764.3513	483.8339	376.6435
ENSMUSG00000023307.14	Marchf5	-0.79801	1.33E-09	4.42E-06	1576.899	1562.103	1258.858	1122.591	736.3116	836.6446	717.4566	881.5696
ENSMUSG00000032380.9	Dapk2	-0.60904	2.54E-08	7.05E-05	862.939	809.4413	720.3003	722.9687	465.3888	522.6205	518.3935	532.5217
ENSMUSG00000027035.10	Cers6	0.491419	1.39E-07	0.00029	801.0679	738.1366	693.6225	783.4924	1100.312	1110.004	998.7715	1047.889
ENSMUSG00000002489.16	Tiam1	1.254034	1.24E-07	0.00029	148.9791	151.8528	221.7591	94.51655	432.1468	390.8358	366.3314	270.7358
ENSMUSG00000026158.11	Ogfrl1	0.915859	5.34E-07	0.00099	257.2535	175.621	201.7508	217.2222	309.1511	382.5522	499.7313	416.1724
ENSMUSG00000030775.11	Trat1	1.132414	7.05E-07	0.001175	104.204	195.4279	118.3827	174.9385	427.1605	313.2711	282.0061	283.4149
ENSMUSG00000042284.10	Itga1	-1.06798	8.49E-07	0.001287	1407.567	1011.472	1290.538	927.7545	367.3247	396.8602	761.6928	678.7041
ENSMUSG00000051354.14	Samd3	0.551544	1.10E-06	0.001526	696.0498	682.6773	593.5808	562.1247	952.385	930.0234	1002.227	833.0907
ENSMUSG00000026478.14	Lamc1	-0.48923	1.21E-06	0.001543	1046.924	1155.402	1160.484	1140.002	688.1106	813.2999	875.7394	815.1908
ENSMUSG00000001025.8	S100a6	-0.46416	1.39E-06	0.001543	4684.293	4381.283	4573.573	4724.169	2787.347	3221.571	3513.326	3774.639
ENSMUSG00000039153.17	Runx2	-0.44651	1.34E-06	0.001543	2430.069	2250.062	2557.733	2266.739	1534.121	1680.067	1880.04	1863.826
ENSMUSG00000030257.16	Srgap3	-0.63202	2.29E-06	0.002381	694.4217	777.7503	795.3316	691.4632	360.6763	500.0288	525.9966	507.1635
ENSMUSG00000026080.13	Chst10	-0.60746	2.47E-06	0.002423	636.621	670.7932	650.2711	571.2448	373.9732	376.5277	494.2018	407.2225
ENSMUSG00000039521.13	Foxp3	-2.25837	2.69E-06	0.002495	70.82612	34.33193	61.69239	126.8512	13.29682	7.530554	15.8974	24.61235
ENSMUSG00000047632.11	Fgfbp3	-2.32848	4.08E-06	0.003581	78.15297	97.71396	70.02919	44.771	18.28313	2.259166	21.42693	16.40823
ENSMUSG00000007891.16	Ctsd	-0.46937	6.58E-06	0.005487	4957.015	6001.486	6154.232	5158.614	3561.887	4171.927	4568.775	3764.198
ENSMUSG00000055027.17	Smyd1	-0.71755	7.68E-06	0.005894	344.3615	470.0834	430.1793	329.9788	212.7492	226.6697	255.7408	254.3276
ENSMUSG00000026605.14	Cenpf	0.646278	8.13E-06	0.005894	406.2326	293.1419	348.4786	353.1934	613.316	461.623	593.0422	536.2509
ENSMUSG00000007872.3	Id3	1.143465	8.12E-06	0.005894	141.6522	132.0459	81.70073	74.61833	289.2059	266.5816	230.1667	170.049
ENSMUSG00000032012.9	Nectin1	5.130438	1.03E-05	0.007148	0	2.640918	0	0	13.29682	18.82639	25.57408	20.13738
ENSMUSG00000037606.18	Osbpl5	-0.54848	1.13E-05	0.007533	784.786	757.9434	895.3733	690.6341	455.4162	579.8527	580.6007	510.1469
ENSMUSG00000053044.8	Cd8b1	-0.3947	1.28E-05	0.008224	13382.07	15721.38	15032.93	13452.03	9643.521	10554.07	11804.17	11780.36

ENSMUSG00000019539.11	Rcn3	-0.95306	2.22E-05	0.013688	213.2925	239.0031	241.7675	146.7494	84.76725	128.0194	118.8849	96.95773
ENSMUSG00000096780.7	Tmem181b-ps	0.830108	2.80E-05	0.016643	141.6522	134.6868	123.3848	123.5348	315.7996	217.633	212.1957	194.6613
ENSMUSG00000054404.13	Slfn5	0.599	3.00E-05	0.017253	1717.737	1386.482	1290.538	1285.093	1996.186	1656.722	2585.056	2368.006
ENSMUSG00000033910.13	Gucy1a1	1.195427	3.79E-05	0.0207	131.8831	81.86846	96.70698	119.3893	335.7448	333.6036	183.1657	141.7075
ENSMUSG00000109251.1	E230032D23Rik	3.496131	3.85E-05	0.0207	0	0	1.667362	11.6073	64.82201	40.66499	22.80931	33.56229
ENSMUSG00000030844.11	Rgs10	0.628368	4.40E-05	0.022943	218.9911	166.3778	176.7403	214.735	314.1375	280.8897	304.1242	312.5022
ENSMUSG00000001020.8	S100a4	-0.84899	5.13E-05	0.024458	257.2535	163.7369	273.4473	254.5314	147.9272	105.4278	129.2528	146.9283
ENSMUSG00000041515.10	Irf8	0.579126	5.06E-05	0.024458	336.2206	324.8329	356.8154	436.1027	571.7634	476.6841	615.8515	514.6218
ENSMUSG00000033952.14	Aspm	0.669012	4.97E-05	0.024458	324.0092	262.7713	303.4598	234.6332	538.5213	368.9972	409.8765	476.5846
ENSMUSG00000094051.6	Ighv1-36	6.685472	5.45E-05	0.02526	0.814093	2.640918	0	0.829093	0	206.3372	39.39791	196.153
ENSMUSG00000060131.11	Atp8b4	-0.68394	5.80E-05	0.025444	3104.138	2787.489	3466.445	3284.865	1379.545	1539.998	2560.173	2379.194
ENSMUSG00000032020.15	Ubash3b	-0.40179	5.65E-05	0.025444	2082.451	2378.147	2451.022	2381.983	1492.568	1845.739	1744.567	1933.188
ENSMUSG00000067591.12	Klra3	-1.1724	6.01E-05	0.025707	239.3435	328.7943	336.8071	169.964	78.11884	105.4278	191.46	96.21191
ENSMUSG00000015314.10	Slamf6	0.617108	6.32E-05	0.026331	3746.458	3232.484	2606.086	2585.94	5574.693	5062.792	4701.483	3342.804
ENSMUSG00000024538.8	Ppic	-1.07672	6.98E-05	0.027451	144.9086	132.0459	138.391	111.0984	54.8494	48.19555	51.83935	93.22859
ENSMUSG00000022797.16	Tfrc	0.49548	7.04E-05	0.027451	800.2538	629.8589	817.0073	708.045	988.9512	912.7032	1210.276	1049.381
ENSMUSG00000015243.4	Abca1	1.028474	7.08E-05	0.027451	262.1381	128.0845	110.0459	155.0403	452.092	328.3322	278.5501	294.6023
ENSMUSG00000097519.2	4930558J18Rik	5.246586	8.39E-05	0.031808	0.814093	0	0	0	19.94524	17.32027	8.294296	5.96663
ENSMUSG00000031004.8	Mki67	0.554134	8.74E-05	0.032389	4720.928	2993.48	4050.022	4282.263	7175.298	5100.444	6120.499	5184.255
